# The hierarchical structure of fear of personal death: from the general factor to specific forms

**DOI:** 10.1186/s41155-020-00152-x

**Published:** 2020-07-20

**Authors:** Jarosław Jastrzębski, Radosław Rogoza, Sławomir Ślaski

**Affiliations:** 1 Anthropological Psychology Center, Faculty of Christian Philosophy, Institute of Psychology, Cardinal Stefan Wyszyński University in Warsaw, Wóycickiego 1/3 (budynek 14), 01-938 Warsaw, Poland; 2 Department of Intercultural Psychology, Faculty of Christian Philosophy, Institute of Psychology, Cardinal Stefan Wyszyński University in Warsaw, Wóycickiego 1/3 (budynek 14), 01-938 Warsaw, Poland; 3 Department of Clinical Psychology, Faculty of Christian Philosophy, Institute of Psychology, Cardinal Stefan Wyszyński University in Warsaw, Wóycickiego 1/3 (budynek 14), 01-938 Warsaw, Poland

**Keywords:** Death anxiety, Fear of personal death, Bi-factor analysis, Hierarchical structure, Exploratory structural equation models

## Abstract

**Purpose:**

In the present study, we aimed to integrate unidimensional and multidimensional perspectives of the construct of the fear of personal death (FOPD). It has been assumed that (a) there is one general factor of FOPD, reflecting the unidimensional perspective and that (b) FOPD assumes a hierarchical structure reflecting the multidimensional perspective.

**Methods:**

We administered the Death and Dying Anxiety Inventory (FVTS, Ochsmann, 1993) to 1217 Polish participants (602 women and 615 men) aged between 18 and 89 (*M*_Age_ = 31.13; SD_Age_ = 12.65).

**Results:**

The results of the bi-factor model of the confirmatory factor analysis proved the existence of a FOPD general factor. Using the bass-ackwards approach, we provided evidence on the hierarchical structure of FOPD, which stresses that specific types of FOPD distinguished in the FVTS, which, on a higher level, make up the factors of threats to self-fulfilling existence, threats to well-being and threats of physical destruction, which in turn depend on the subject’s perspective: the physical self and/or the symbolic self.

**Conclusion:**

The current study demonstrates that unidimensional and multidimensional approaches to FOPD do not necessarily exclude one another. The unidimensional approach to FOPD seems to be most appropriate for studying the intensity of FOPD, while the multidimensional approach seems to be more suitable for studying the individual differences in how people give meaning to FOPD.

The fear of personal death (FOPD) is defined as an unpleasant emotional experience caused by thinking about one’s own or another person’s death (Nyatanga & de Vocht, 2006). It is a consequence of a form of awareness specific to humans that enables them to learn that they are mortal—that their life is limited in time (Yalom, 2008a). Thus, the FOPD is a common and unavoidable experience, and the way a person lives and develops depends on the way they cope with that experience (Becker, 1973/2015; May, 1989, 1994; Yalom, 2008b).

Confronting the inevitability of one’s own death constitutes an important motive of human existence (Menzies, 2012). Yalom (2008b) argues that, generally, one’s whole life is an effect of the mechanisms that offer protection from experiencing the consequences of the FOPD. Most people use adaptive strategies to deal with the FOPD; however, under certain circumstances (e.g. real threat to one’s life or health or that of their loved ones), the lack of an adequate defence strategy might take the shape of pathology (Kastenbaum, 2000; Yalom, 2008b). Yalom (2008b) argues that those defensive mechanisms, which successfully diminish strong fear, simultaneously diminish development and in effect lead to a restricted and unsatisfactory life. Such fear might be nonspecific, to the extent that one has to project it on to more specific objects (e.g. fear of a heart attack). This is why the FOPD is considered a primary type of fear, the base of other fears, and is a contributor to the development of different mental disorders (Arndt, Routledge, Cox, & Goldenberg, 2005; Furer & Walker, 2008; Iverach, Menzies, & Menzies, 2014; Strachan et al., 2007).

For this reason, expanding the knowledge of the FOPD is an important research objective. It is possible to pursue this objective by investigating the determinants, functions or development of the FOPD (Feifel & Nagy, 1981; Yalom, 2008b); however, the most important issue is the study of the structure of FOPD, which will provide researchers with a common ground for interpreting their research results. This is precisely the issue addressed in the present paper.

## One or many fears of death?

Initially, the FOPD was understood as a unidimensional phenomenon (Boyar, 1964), and the first measuring instruments were also unidimensional (e.g. the Fear of Death Scale, Boyar, 1964; the Death Anxiety Scale–Modified, Chow & Henry, 2017; the Death Anxiety Scale, Templer, 1970). They allowed the intensity of the FOPD to be measured but did not measure individual differences in the meanings people attributed to death; consequently, the possibility of understanding the complex structure of the FOPD was limited (Kastenbaum & Costa, 1977). Another problem with the unidimensional scales was the lack of information about their factorial structure. They were composed of items describing various aspects of the FOPD; however, users were unable to specify which of these aspects was actually measured, giving rise to misunderstandings in the interpretation of the obtained data (Wittkowski, 2001).

Later scholars began to highlight the fact that there are many reasons to feel afraid of death (Kastenbaum, 2000; Kastenbaum & Costa, 1977). People usually fear that (a) their death will sadden their family and friends, (b) death will put an end to all their plans and goals, (c) they will no longer have a chance to experience anything, (d) the process of dying will be painful, (e) they do not know if there is an afterlife and (f) they do not know what will happen to their body after death (Diggory & Rothman, 1961). This view inspired a large number of instruments measuring the different types of FOPD (e.g., the Fear of Personal Death Scale, Florian & Kravetz, 1983; the Multidimensional Fear of Death Scale, Hoelter, 1979; the Collett and Lester Fear of Death Scale, Lester, 2004), but according to Ochsmann (1993) and Wittkowski (2001), their main shortcoming is the lack of clear criteria for identifying the sources of FOPD. As a result, the thanatological literature is full of diverse classifications in terms of content, which postulate a structure of the FOPD consisting of four (Lester, 2004), five (Conte, Weiner, & Plutchik, 1982), six (Florian & Kravetz, 1983) or eight (Hoelter, 1979) dimensions. According to Lester (2004), the types of FOPD are the (a) fear of one’s own death, (b) fear of other people’s death, (c) fear of one’s own dying process and (d) fear of other people’s dying process. Both Florian and Kravetz (1983) and Mikulincer and Florian (2008) identify the types of FOPD based on the consequences of death (a) for one’s own mind and body (intrapersonal), (b) for one’s family and friends (interpersonal) and (c) for the transcendent nature of the human self (transpersonal). For Ochsmann (1993), the basis of the classification of the FOPD types is the meaning of death, resulting from a particular way of perceiving a human being (a) as part of nature (death as decay and decomposition processes), (b) as a psychosocial being (death as the loss of identity and bonds with close others) and (c) as a being capable of self-reflection (death as the loss of the possibility of experiencing anything). The diversity of all these classifications suggests that the structure of the FOPD is a complex issue.

Advocates of the unidimensional approach question the need for this kind of classification on the grounds that it is not certain whether they offer a better understanding of human behaviour and that they can obscure the picture (Łukaszewski, 2010). In their opinion, the study of its consequences on everyday human life is more important than identifying the specific sources of the FOPD, as in the research based on the terror management theory (Solomon, Greenberg, & Pyszczynski, 2015). Thus, it can be concluded that regardless of how precisely we define the object of the FOPD, two central issues remain the same: the fact that we experience fear and the fact that in all cases it concerns death—the fragility of human life. By contrast, Yalom (2008b) believes that death anxiety is a mixture of distinct, more or less specific, fears, amongst which the central issue is the fear of personal annihilation—the fear of ceasing to exist. In this view, it is legitimate to suspect that the two approaches (unidimensional and multidimensional) do not exclude each other but allow a better understanding of different portions of human existence in which the FOPD performs an important function. Within the structure of the FOPD, it is possible to distinguish both a general dimension and a number of specific types that make it up; the aspects that should be the focus are determined by the objectives and the accuracy of the instruments used.

There are two approaches to studying the multidimensional structure of a measure. The first is the higher-order model in which the overall score is the result of the lower-order factors. In such an approach, the overall score is the result of the common variance of all the specific factors as it forces a primary trait to be a domain specific factor and it would be unclear to test both the overall score and the specific factors (Reise, Moore, & Haviland, 2010). The second approach to test the multidimensionality of the measure is the bi-factor approach (see Blasco-Belled, Rogoza, Torrelles-Nadal, & Alsinet, 2019 for an empirical illustration of the bi-factor model utility). In this case, the overall score is not the result of the specific scales’ common variance but rather the result of the items’ shared variance being hypothesised to have something in common; thus, the role of domain specific factors that are independent of the general factor may be studied (Chen, West, & Sousa, 2006). It also enables one to analyse the additional common variance amongst a cluster of items that explains something specific in addition to what was explained by the bi-factor. The bi-factor model thus appears to be ideally suited to analyse the construct-relevant multidimensionality (Reise, Scheines, Widaman, & Haviland, 2013). In summary, the bi-factor can be understood as a result of the commonality between the items (general variance), which is an addition to the item domain specificity (group variance; Rodriguez, Reise, & Haviland, 2016), and each of these is hypothesised to introduce a new quality leading to a better understanding of the analysed constructs.

However, the question of links between the different types of FOPD is still pending. A comparison of the obtainable classifications suggests, firstly, that certain types of FOPD (e.g., fear of life after death, fear of the process of dying or anxiety about the body after death) occur independently of the adopted identification criterion, and secondly, that the types of FOPD that are distinguished as independent in one classification may be included in other classifications in the range of more broadly defined types of FOPD (Ochsmann, 1993). Such observations suggest a hierarchical structure of the FOPD.

## Present study

As there are discrepancies in the literature on the meaning and structure of the FOPD, the aim of the present study was to examine it in a more nuanced way, with the *Furcht vor Tod und Sterben Fragebogen* (Death and Dying Anxiety Inventory, FVTS) as an empirical illustration. More specifically, we hypothesised that (a) the inconsistencies and contradictory results in previous research were due to the existence of the general fear of death factor, which could be meaningfully differentiated into specific forms of the FOPD; and (b) the FOPD would turn out to have a hierarchical structure. To test whether the structure of the FVTS comprised the general fear of death in addition to more specific forms of FOPD, we performed a bi-factor confirmatory factor analysis. To test whether the structure of the FOPD is hierarchical in nature, we ran a series of exploratory structural equation models as suggested in Goldberg’s (2006) top-down procedure.

## Method

### Participants and procedure

The study was conducted in central, eastern and southern Poland on a group of 1217 people (602 women and 615 men) between 18 and 89 years of age (*M*_Age_ = 31.13; SD_Age_ = 12.65). The sample consisted of people in the following age ranges: between 18 and 23 years old (*M*_Age_ = 20.08; SD_Age_ = 1.79), constituting 35%; between 24 and 40 years old (*M*_Age_ = 30.76; SD_Age_ = 4.98), constituting 38%; between 41 and 59 years old (*M*_Age_ = 49.93; SD_Age_ = 4.95), constituting 16%; and over 60 years old (*M*_Age_ = 65.61; SD_Age_ = 5.74), constituting 3%. There was no data on age for 8% of the sample. Twenty-nine per cent of participants had a secondary education, 23% had an elementary education (17% of them were secondary school students) and 23% had a higher education. We did not obtain information on 25% of the participants’ education level.

Data were collected using the paper-pencil method. Participants completed the questionnaire both individually and in small groups. Some of the participants were psychology students at the Cardinal Stefan Wyszyński University in Warsaw and completed the questionnaire during optional classes in small groups of 15–20 people. Then, under the guidance of a researcher, they invited more participants to participate in the study, which initiated the recruitment process using the snowball method. Before responding to the questionnaire, all participants received information stating that the study concerns the fear of death, that participation is voluntary and anonymous and that the results will only be used for scientific purposes.

All procedures performed in studies involving human participants were in accordance with the ethical standards of the institutional and/or national research committee and with the 1964 Helsinki Declaration and its later amendments or comparable ethical standards.

### Measures

The FVTS (Ochsmann, 1993) is a 48-item instrument measuring six types of death anxiety: *fear of encountering death* manifests itself in a fear of contact with the dead and the dying; *fear of mortality* is a fear that death will make it impossible to take care of others, that one will not achieve the goals one has set for oneself, that one will not fulfil one’s obligations and that death makes it impossible to experience anything; *fear of the end of one’s life* refers to anxiety connected with the awareness of passing and the inevitability of death; *fear of physical destruction* is the anxiety about what will happen to the body after death; *fear of life after death* manifests itself in uncertainty about further, posthumous existence and in the perception of life after death as a terrifying reality because it is unknown; and *fear of the process of dying* is a fear of the suffering and pain that accompanies dying.

The questionnaire items were translated from the original into Polish, and then back-translated into German by a German language teacher. The FVTS items, along with their Polish translations, are available in the Appendix. The participants’ task was to rate on a 3-point scale (*true* = 2; *hard to say*, *neither truth nor false* = 1; *false* = 0) whether FVTS items were true for them. In the original version, the reliability coefficients for particular scales ranged from *α* = 0.73 (for the fear of encountering death scale) to *α* = 0.88 (for the fear of the process of dying scale).

Our choice of the FVTS questionnaire for the present study was dictated by the fact that it seems to overcome the shortcomings of the measurements described above. It was designed as a multidimensional measure, but it also allows the overall score to be computed. Its items were selected according to their content reflecting the typology of thanatic anxiety as stated by Kastenbaum and Aisenberg (1972), which combines different approaches to classifying the types of FOPD.

## Results

### Structural validity of the FVTS

As a preliminary step of the analyses, we ran Horn’s (1965) parallel analysis on 5000 randomly generated correlation matrices to assess the underlying factorial structure of the measure (Table 1). The average eigenvalues generated from parallel analysis were higher than those generated from the sample correlation matrix up to the seventh factor. However, the first eigenvalue in the sample correlation matrix was over three times higher (10.11) than the second eigenvalue (2.96), which suggests the existence of a general factor saturated by all the remaining factors. Moreover, the explained common variance of the general factor stood at 0.39, indicating that specific factors also make a unique contribution to the general level of fear of death. In such circumstances, instead of analysing the higher-order factors, implementing a bi-factor appears to be preferable (Reise, 2012).

**Table 1:** Standardised factor loadings from the bi-factor model of the FVTS

Item	General death anxiety	F1	F2	F3	F4	F5	F6
1	0.64		0.06				
2	0.28				0.37		
3	0.67					0.43	
4	0.48						0.16
5	0.31			1.00			
6	0.22				0.57		
7	0.49			0.06			
8	0.27				0.44		
9	0.45	0.67					
10	0.37	0.44					
11	0.41						0.56
12	0.79			0.18			
13	0.46	0.62					
14	0.54		0.61				
15	0.38	0.70					
16	0.30				0.52		
17	0.72					0.49	
18	0.50	0.60					
19	0.58						0.59
20	0.46		0.50				
21	0.41	0.53					
22	0.74			0.21			
23	0.45					0.32	
24	0.41		0.47				
25	0.50		0.53				
26	0.62		0.21				
27	0.56			−0.04			
28	0.29						0.38
29	0.39	0.40					
30	0.79			0.06			
31	0.24				0.83		
32	0.45						0.56
33	0.51					0.40	
34	0.52						0.62
35	0.38				0.58		
36	0.48		0.54				
37	0.45					0.48	
38	0.69					0.26	
39	0.32				0.89		
40	0.71					0.27	
41	0.50						0.75
42	0.48	0.68					
43	0.80		0.06				
44	0.22			0.46			
45	0.52						0.33
46	0.41					0.25	
47	0.32				0.43		
48	0.77			−0.02			

*F1* fear of encountering death; *F2* fear of mortality; *F3* fear of the end of one’s life; *F4* fear of physical destruction; *F5* fear of life after death; *F6* fear of the process of dying

Because the response scale offered only three categories, we treated our data as categorical (Rhemtulla, Brosseau-Liard, & Savalei, 2012) and ran the bi-factor confirmatory factor analysis on a polychoric correlation matrix using the WLSMV estimator. We conducted the analysis using Mplus version 7.2 (Muthén & Muthén, 2012).

The analysed model^1^ was well fitted to the data (*χ*^2^_(1032)_ = 3164.52, *p* < 0.001; CFI = 0.936; RMSEA = 0.041, 90% CI [0.040, 0.043], *p* = 1.00). The bi-factor was significantly loaded by all of the items, and the mean strength of the factor loadings was optimal (*M* = 0.48) to conclude that the saturation of the bi-factor is important for FVTS measurement. The differentiation of the grouping factors was also important for the FVTS structure, as the mean strength of the factor loadings was higher than 0.30 for all scales except the fear of the end of one’s life, and only two other scales—the fear of mortality (0.37) and the fear of life after death (0.36)—had a mean factor loading strength below that of the bi-factor. The reliability estimates of the scales distinguished were as follows: general death anxiety *ω* = 0.93; F1*—*fear of encountering death *ω* = 0.83; F2—fear of mortality *ω* = 0.76; F3—fear of the end of one’s existence *ω* = 0.75; F4—fear of physical destruction *ω* = 0.78; F5—fear of life after death *ω* = 0.80; and F6—fear of the process of dying *ω* = 0.79. Thus, it can be concluded that the bi-factor measurement model with specific death anxiety factors is an adequate and reliable structural representation of the FVTS.

## The hierarchical structure of the fear of personal death

**Fig. 1:**
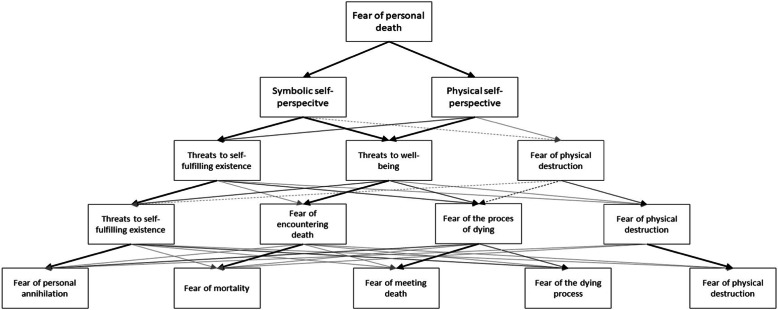
Hierarchical structure of the fear of personal death. Arrows represent significant correlations between factors higher than 0.40. The strongest correlations have been bolded. Note. Thick black arrow: correlation > 0.70; black arrow: correlation > 0.50; grey arrow: correlation > 0.30; dotted line: negative correlation

Previous analyses (factor loadings are presented in Table 1) demonstrated the existence of a general fear of death influencing all of the specific forms of the FOPD; however, they did not demonstrate how general FOPD differentiates into more specific forms. To assess the hierarchical structure of the FOPD, we used Goldberg’s (2006) top-down procedure, that is, we ran six independent exploratory structural equation models varying the number of factors (from an unidimensional model to a six-factor model), extracted the factor scores from each model and correlated these factor scores across models (e.g. factor scores from the two-factor model were correlated with the scores from the three-factor model, which in turn were correlated with the four-factor model, and so on). The results of the analysis are presented in Fig. 1.

Initially, we intended to identify six levels of the hierarchy of the FOPD, which is the same number of types of fear of death distinguished in the FVTS questionnaire, but the six-factor solution did not produce the expected results. The items that make up the FVTS fear of the end of one’s life scale loaded on several different factors in a manner that made it impossible to make theoretical interpretations. The first level of the hierarchy is represented by one factor, corresponding to general FOPD. On the second level, this factor splits into two factors, one of which is saturated with items concerning the fear of physical destruction and the fear of encountering death, while the other is saturated with items concerning the fear of mortality, the fear of the end of one’s existence, the fear of life after death and the fear of the process of dying. The analysis of the content of items suggests that they correspond to two perspectives of the self as described by Becker (1973/2015): the *physical self-perspective* and the *symbolic self-perspective*. On the third level, from the physical self-perspective, a factor comprising items associated with the fear of physical destruction scale splits off. Additionally, a group of items split off from the physical self-perspective and symbolic self-perspective, and their contents concern the fear of encountering death and the fear of the process of dying. The analysis of the contents of these items suggests that we deal with fear whose source is the perception of death in terms of threats to psychophysical well-being (a fear of pain, suffering and slow death). Moreover, from the symbolic self-perspective, a factor splits off whose essence is a fear of death perceived in terms of threats to self-fulfilling existence. On the fourth level, the factors of the fear of physical destruction and threats to self-fulfilling existence remain unchanged, while the threats to well-being factor split into factors whose contents in the FVTS correspond to the fear of encountering death and fear of the process of dying scales. On the fifth level, the factors of fear of physical destruction, fear of the process of dying and fear of encountering death remain unchanged. Threats to self-fulfilling existence split into two factors: one is made up of items corresponding to the FVTS fear of mortality scale, while the other comprises items corresponding to the FVTS fear of the end of one’s existence and fear of life after death scales. According to existential psychologists (e.g. May, 1989, 1994; Yalom, 2008b), this factor corresponds to the fear of personal annihilation.

## Discussion

Although the multidimensional approach prevails in the current research on FOPD, the structure of FOPD remains an unresolved issue (Łukaszewski, 2010). The review of unidimensional approaches and the existing classifications prompted us to formulate the hypothesis that (a) apart from specific forms of fear of death there is a general thanatic anxiety factor and that (b) the structure of fear of death is hierarchical.

Using bi-CFA, we demonstrated the existence of a general FOPD factor, which was significantly saturated with the types of fear of death distinguished in the FVTS. Moreover, the tested structure appeared to be invariant across gender. This means both approaches to FOPD—unidimensional and multidimensional—are equally valid, provided that they are used to explain an appropriate area of research. While the measurement of the general factor makes it possible to study the level and psychological consequences of the FOPD in everyday human functioning (e.g. research based on the terror management theory (TMT); Solomon et al., 2015), focus on the diversity of meanings and forms of the FOPD is necessary for a complete understanding of the complexity of its structure (Kastenbaum, 2000).

We gained insight into this complex structure of the meanings of the fear of death by examining a series of ESEM models (Goldberg, 2006), and the results of this examination confirmed the hierarchical structure of the FOPD. None of the classifications presented in the introduction (Florian & Kravetz, 1983; Lester, 2004; Ochsmann, 1993) were fully reflected in the model of the hierarchical structure of the FOPD that we obtained. On the first level, we observed the existence of a general factor, which can be called the FOPD. It brings together the specific reasons for which people are afraid of death (Yalom, 2008b). On the second level, the factors seem to reflect the duality of human nature described by Becker (1973/2015), in which the physical self, inevitably a transitory and mortal being, stands in contrast to the symbolic self, which seeks ways to defy mortality. The first factor (physical self-perspective) is saturated with items whose contents concern the fear of facing death (e.g. “The sight of a dead body would be something terrible for me”) and the fear of physical destruction (e.g. “It makes no difference to me what will happen to my body after death”). The second factor (symbolic self-perspective) combines items representing the remaining four dimensions distinguished in the FVTS (e.g. “I am afraid I could die without achieving my life goals”; “It worries me to think that death means the end of my existence”; “It worries me that I don’t know what is going to happen after death”; “I am not worried at the thought that dying can be very painful”). To a limited extent, these factors can be interpreted in terms proposed by Florian and Kravetz (1983) as intrapersonal and transpersonal consequences of death. To some extent, this distinction also corresponds to Ochsmann’s (1993) proposal in which the two factors reflect two different meanings that people attribute to death according to how they perceive the human being: as a being staying close to nature, for whom death means decomposition and decay, or as a non-material social being capable of self-reflection, for whom death involves a loss of identity, bonds, the possibilities of experiencing anything and the possibilities of self-realisation.

On the level of three factors, apart from the two factors that were a combination of several types of the FOPD, we observed a factor corresponding to the FVTS dimension of the fear of physical destruction. This factor remains unchanged on all further levels of the hierarchical structure of the FOPD. The factors of this level seem to correspond to the threats that the person anticipates depending on the perspective adopted: the physical or symbolic self. According to Yalom (2008b), the anticipation of one’s own death may work as a critical situation which, depending on the perspective the person adopts (the physical or symbolic self), can be considered in terms of (a) threat/loss or (b) challenge, just like the stressful situation in Lazarus’s (1999) stress theory. Consequently, the first factor in the three-factor solution, associated with the physical self-perspective, is the outcome of perceiving death in terms of a threat of losing one’s body and corporeal identity. It is saturated with items making up the FVTS fear of the physical destruction dimension. The second factor on the third level is associated with perceiving death as a threat both within the physical self-perspective and in the symbolic self-perspective. This factor is saturated with items concerning the fear of encountering death and the fear of the process of dying. Their content pertains, on the one hand, to the anticipation of threats to the sense of uniqueness—the belief that death does not concern us (Yalom, 2008b)—and, on the other hand, to the anticipation of threats to human psychophysical well-being (e.g. pain, suffering, slow death). This dimension can therefore be referred to as threats to well-being. The third factor on the third level is associated with perceiving death as a threat from the symbolic self-perspective. It is made up of items concerning the fear of mortality, the fear of the end of one’s existence and the fear of life after death. Their content seems to express what Yalom (2008b) calls a fear of personal annihilation—a fear that death will prevent the fulfilment of plans and goals and that one will cease to exist. This dimension can therefore be referred to as threats to self-fulfilling existence. This is what Yalom (2008b) considers to be the essence of the FOPD. In his opinion, confronting these aspects of death makes a person aware that existence cannot be put off until later, makes him or her turn away from trivial preoccupations, creates more depth and a completely different perspective to life and promotes development.

On the level of four factors, apart from the fear of physical destruction, two further dimensions of the FVTS manifest themselves: the fear of the process of dying and the fear of encountering death (which made up the threats to well-being dimension on the third level). The threats to the self-fulfiling existence dimension remain unchanged.

On the fifth level, there are four factors, corresponding to the forms of the FOPD distinguished in the FVTS (i.e. the fear of physical destruction, the fear of the process of dying, the fear of encountering death and the fear of mortality), as well as a fifth factor, the fear of personal annihilation, made up of items associated with the fear of the end of one’s life and the fear of life after death. This corresponds to what May (1989, 1994) refers to as a fear of losing oneself and vanishing into nothingness. This type of death anxiety—like none other—turns a person towards their own (symbolic) self, becomes a challenge for them, forces them to engage in life and motivates them to develop and discover their abilities, thereby increasing their chance of finding purpose and meaning in life (Frankl, 2011).

In summary, the results of our study contribute to the broadening of knowledge on FOPD in two ways. First, our analysis made it possible to integrate two approaches to the FOPD described in the literature: unidimensional and multidimensional. We found that in the structure of the FOPD, measured by means of the FVTS, there is one general dimension that can be identified and that it is significantly saturated with more specific forms of the FOPD. This means the two approaches are equally valid, provided that they are applied to explain an appropriate area of research. In the investigation of individual differences concerning the meanings people attribute to death, it is necessary to measure specific forms of the FOPD, but when investigating the level and consequences of death anxiety the general factor is sufficient. Second, the hierarchical analysis revealed that, apart from the general factor, confrontation with the awareness of one’s own death can be considered on at least three levels: (a) specific types of fear of personal death, which stem from (b) perceiving death in terms of threats and (3) the inevitably transitory, mortal physical self or the symbolic self that seeks ways to defy death. On the one hand, these findings provide researchers with a common ground for interpreting results, depending on the level of generality of their analyses; on the other hand, they call for further research, on the basis of which the hierarchical model of the structure of FOPD could be confirmed and elaborated.

## Conclusions

The results allow for the integration of two approaches to study the FOPD. Unidimensional and multidimensional approaches to study the FOPD appear to be theoretically equivalent when used for specific reasons. For example, using a unidimensional score appears to be sufficient to study the intensity and consequences of the FOPD. In turn, using a multidimensional approach appears to be better suited to study the individual differences in how people ascribe meaning to the FOPD. Given the hierarchical structure of the FOPD, it is recommended that at least three dimensions of FOPD are studied: specific types of FOPD distinguished in the FVTS, which, on a higher level, make up the factors of threats to self-fulfilling existence, threats to well-being and threat of physical destruction, which in turn depend on the subject’s perspective: the physical self and/or the symbolic self.

## Supplementary information

**Additional file 1.** Appendix: The Polish form of the FVTS.

## Data Availability

The datasets used and/or analysed during the current study are available from the corresponding author on reasonable request.

## Abbreviations

FOPD: Fear of personal death
FVTS: Furcht vor tod und sterben (fear of death and dying)
WLSMV: Weighted least squares with means and variances adjusted
CFI: Comparative fit index
RMSEA: Root mean square error of approximation
CFA: Confirmatory factorial analysis
TMT: Terror management theory

## Footnotes

1 Additionally, we have examined whether this structure is invariant across gender. We have found support for full scalar invariance, as compared to the unconstrained configural model. The fit indices of the compared invariance models were as follows: Configural: χ^2^_(2064)_ = 3942.95; CFI = 0.944; RMSEA = 0.039; Scalar: χ^2^_(2194)_ = 3977.74; CFI = 0.947; RMSEA = 0.037.
