# Supplementary material for: The hierarchical structure of fear of personal death: from the general factor to specific forms
Source: Psicol Reflex Crit. 2020 Jul 20;33:16. doi: 10.1186/s41155-020-00152-x (PMC7371767; doi:10.1186/s41155-020-00152-x)
Supplement: Supplementary file 1 — Additional file 1. Appendix: The Polish form of the FVTS. [file 41155_2020_152_MOESM1_ESM.docx]

**Appendix: The Polish form of the FVTS:**

# **FVTS (PL)**

Instrukcja:

Kwestionariusz ten zawiera szereg stwierdzeń dotyczących śmierci i umierania. Bardzo proszę, abyś ustosunkował(a) się do nich w sposób jak najbardziej osobisty.

Nie ma tu ani złych, ani dobrych odpowiedzi. Poprawne są tylko te odpowiedzi, które są zgodne z Twoimi własnymi przekonaniami.

Przy każdym stwierdzeniu możesz zakreślić jedną, najbardziej właściwą dla Ciebie odpowiedź. Proszę zakreślić:

**P** (PRAWDA) – jeśli stwierdzenie odnosi się do Ciebie

**?** – jeśli nie potrafisz ocenić, czy to stwierdzenie odnosi się do Ciebie, czy też nie odnosi się.

**F** (FAŁSZ) – jeśli stwierdzenie nie odnosi się do Ciebie.

|  | Śmierć jest dla mnie straszna, ponieważ nie będę mógł(a) już nigdy więcej niczego doświadczyć. | P | ? | F |
| --- | --- | --- | --- | --- |
|  | Nie zależy mi na tym, aby troszczono się o mój grób. | P | ? | F |
|  | Boję się śmierci, ponieważ nie wiem, co ze mną się stanie, jeśli istnieje życie po śmierci. | P | ? | F |
|  | Nie jestem przerażony(a), gdy myślę o śmierci spowodowanej uduszeniem, bądź utonięciem. | P | ? | F |
|  | Myślę, że to dobrze, że moje życie ma kres. | P | ? | F |
|  | Chciał(a)bym, aby po śmierci moje oczy zostały oddane komuś choremu. | P | ? | F |
|  | Gdybym wiedział(a) jak szybko życie upływa, był(a)bym tym bardzo zaniepokojony(a). | P | ? | F |
|  | Nie jest dla mnie istotne, czy zostanę pochowany(a) w drewnianej trumnie, czy metalowej skrzyni. | P | ? | F |
|  | Mógł(a)bym wejść do dowolnego pomieszczenia, w którym ktoś umiera. | P | ? | F |
|  | Opiekowanie się kimś umierającym byłoby dla mnie bardzo trudne. | P | ? | F |
|  | Byłoby dla mnie czymś strasznym, jeśli miał(a)bym umierać powoli. | P | ? | F |
|  | Niepokoi mnie myśl, że śmierć oznacza koniec mojego istnienia. | P | ? | F |
|  | Nie odczuwam lęku przed wejściem do prosektorium. | P | ? | F |
|  | Niepokoi mnie myśl, że mogę nie mieć dość czasu, aby zrobić wszystko to, co bym chciał(a). | P | ? | F |
|  | Dotykanie zmarłego nie byłoby dla mnie problemem. | P | ? | F |
|  | Nie chcę, aby dokonano sekcji moich zwłok. | P | ? | F |
|  | Napawa mnie niepokojem to, że nie wiem, co będzie po śmierci. | P | ? | F |
|  | Znalezienie kogoś zmarłego byłoby dla mnie okropnym przeżyciem. | P | ? | F |
|  | Myśl, że mojemu umieraniu może towarzyszyć duże cierpienie, wywołuje we mnie lęk. | P | ? | F |
|  | Niepokoi mnie myśl, że po śmierci nie będę już mógł(a) troszczyć się o moich bliskich. | P | ? | F |
|  | Mógł(a)bym spać w jednym pokoju ze zmarłym. | P | ? | F |
|  | Nie przeraża mnie myśl, że w końcu muszę umrzeć. | P | ? | F |
|  | Nie wywołuje we mnie niepokoju wyobrażenie, że po śmierci moje czyny poddane zostaną osądowi. | P | ? | F |
|  | Myśl, że umierając zostawię bliskie mi osoby, nie wywołuje we mnie niepokoju. | P | ? | F |
|  | Nie przeszkadza mi to, że mógł(a)bym umrzeć zanim zrobię wszystko to, co chciałem(am). | P | ? | F |
|  | Boję się śmierci, ponieważ sprawi ona ból moim bliskim i przyjaciołom. | P | ? | F |
|  | Często nachodzi mnie myśl, że życie jest właściwie takie krótkie. | P | ? | F |
|  | Nie niepokoi mnie myśl, że umieranie może być bardzo bolesne. | P | ? | F |
|  | Nie sprawiłoby mi trudności odwiedzenie umierającego kolegi/ koleżanki. | P | ? | F |
|  | Fakt, że kiedyś będę martwy(a) nie wywołuje we mnie żadnego lęku. | P | ? | F |
|  | Jestem przeciwny(a) temu, aby moje ciało było wykorzystywane po mojej śmierci w celach naukowych. | P | ? | F |
|  | Nie boję się, że mógł(a)bym długi czas chorować zanim umrę. | P | ? | F |
|  | Dręczy mnie pytanie o to, czy istnieje życie po śmierci. | P | ? | F |
|  | Obawiam się śmierci z powodu możliwości długiego umierania. | P | ? | F |
|  | Nie ma dla mnie znaczenia, co stanie się z moim ciałem po śmierci. | P | ? | F |
|  | Obawiam się, że mógł(a)bym umrzeć bez osiągnięcia swoich celów życiowych. | P | ? | F |
|  | Obawiam się, że po śmierci nie zaznam spokoju. | P | ? | F |
|  | Myśl, że wraz ze śmiercią moje istnienie mogłoby być na zawsze zakończone wywołuje we mnie lęk. | P | ? | F |
|  | Nie mam nic przeciwko temu, aby moje ciało miało kiedyś służyć studentom medycyny. | P | ? | F |
|  | Nie odczuwam jakiegokolwiek lęku przed tym, co przyjdzie po śmierci. | P | ? | F |
|  | Boję się śmierci poprzedzonej długimi cierpieniami. | P | ? | F |
|  | Oglądanie zwłok byłoby dla mnie czymś strasznym. | P | ? | F |
|  | Chociaż śmierć mogłaby zniweczyć moje plany, wcale jej się nie obawiam. | P | ? | F |
|  | Chciał(a)bym być nieśmiertelny(a). | P | ? | F |
|  | Nie mam jakiejś szczególnej obawy przed zachorowaniem na raka. | P | ? | F |
|  | Nie obawiam się stanąć kiedyś przed moim Stwórcą. | P | ? | F |
|  | Myśl, że moje ciało po śmierci mogłoby zostać spalone, nie przeraża mnie. | P | ? | F |
|  | W ogóle nie obawiam się umierania. | P | ? | F |
